# Air Contamination in Different Departments of a Tertiary Hospital. Assessment of Microbial Load and of Antimicrobial Susceptibility

**DOI:** 10.3390/biomedicines8060163

**Published:** 2020-06-17

**Authors:** Athanasios Tselebonis, Evangelia Nena, Maria Panopoulou, Christos Kontogiorgis, Eugenia Bezirtzoglou, Theodoros Constantinidis

**Affiliations:** 1 Laboratory of Hygiene and Environmental Protection, Medical School, Democritus University of Thrace, 68100 Alexandroupolis, Greece; atselemp@med.duth.gr (A.T.); ckontogi@med.duth.gr (C.K.); empezirt@med.duth.gr (E.B.); tconstan@med.duth.gr (T.C.); 2 Laboratory of Microbiology, Democritus University of Thrace, University General Hospital of Alexandroupolis, 68100 Alexandroupolis, Greece; mpanopou@med.duth.gr

**Keywords:** hospital hygiene, air contamination, nosocomial infection, drug resistance, microbial load

## Abstract

Air contamination in the hospital setting can be a reason for the spread of nosocomial infection among susceptible patients. The aim of this study was to identify bacterial species, and their load and drug resistance, in the air of a tertiary hospital. Air samples were collected on a monthly basis for 12 consecutive months in four different departments of the hospital (Intensive Care Unit (ICU), Internal Medicine Ward (IMW), Surgical Ward (SW), and Neonatal Unit (NU)). In total, 101 samples were collected, out of which 158 Gram-positive (GP) and 44 Gram-negative (GN) strains were isolated. The majority of GP isolates were *Staphylococcus* spp. (*n* = 100). The highest total microbial load was reported in the IMW (*p* = 0.005), while the highest Staphylococcus load was observed in the ICU (*p* = 0.018). GP bacterial load was higher in autumn, while GN load was higher in spring. Regarding drug resistance, four multi-drug-resistant (MDR) strains and one extensively drug-resistant (XDR) strain were isolated in the ICU, two MDR strains and one XDR strain in the SW, one MDR strain in the IMW and one MDR strain in the NU samples. Air in hospital settings is contaminated with various microbes; some of them are MDR, consisting a potential cause of hospital-acquired infection.

## 1. Background

Hospital-acquired infections are a significant health problem globally, and the majority of them, namely 60%, are attributed to drug-resistant pathogens [1,2]. Although hands are considered the primary vector for the transfer of microbes in the hospital setting, contamination of indoor air with pathogens may lead to the spread of nosocomial infections among immune-compromised or other groups of susceptible patients [3]. Airborne pathogens, which mainly originate from patients through cough droplets or skin squamae [3,4,5], can colonize the air throughout patient care settings, and their survival depends on various environmental conditions [6,7]. Surveillance of airborne pathogens in healthcare settings is important not only for academic/epidemiologic purposes but also for the health and safety of patients and healthcare workers. Implementation of control measures can significantly reduce the risk of dissemination of pathogens and the consequent incidence of hospital-acquired infections.

In order to monitor the colonization of air in the hospital environment, a study protocol was designed aiming specifically at identifying bacterial species, measuring their load and seasonal variations as well as their drug resistance in the air of four different settings of a tertiary hospital on a monthly basis for a total period of 12 months.

## 2. Methods

The study was conducted in a tertiary university hospital in NE Greece for a period of 12 months. Air samples were collected on a monthly basis from four departments of the hospital, located in different floors with significant distance from each other, namely, the Intensive Care Unit (ICU), the Neonatal Unit (NU), the Internal Medicine Ward (IMW), and the Surgical Ward (SW).

### 2.1. Air Sampling Methodology

Air sampling was conducted with a Duo SAS Super 360, PBI-International (BIOSCIENCE INTERNATIONAL, Rockville, MD USA), according to the procedure described by the manufacturer. Air flow was conducted through a specially designed nutrient plate with 219 small openings, with a flow rate of 200 lt/min and sampling time of 5 min. The air was targeted on the agar surface of a Petri plate, bearing the proper nutrient mean, i.e., MacConkey agar (Merck KGaA, Darmstadt, Germany) for Gram-negative (GN) pathogens; Pseudomonas agar for *Pseudomonas aeruginosa* (Oxoid Ltd. Thermo Fischer Scientific Inc., UK); Baird–Parker agar for *Staphylococcus aureus* (Oxoid Ltd. Thermo Fischer Scientific Inc., UK); Slanetz–Bartley- for *Enterococcus* spp. and nutrient agar (Oxoid Ltd. Thermo Fischer Scientific Inc., UK) for total microbial load. Incubation was performed under aerobic conditions at 37 °C for 24 h. Microbial load was expressed as colony-forming units per cubic meter of air (CFU/m^3^).

### 2.2. Identification of the Isolates

Bacteria identification at genus and species level was conducted according to standard laboratory techniques. More specifically, it was based on the following: (a) the nutrients, into which colonies were developed, (b) the colonies’ morphology, (c) oxidase reaction for P. Aeruginosa, (d) vatalase and pectinase reaction for *S. aureus*, (e) aesculin for *Enterococcus* spp., (f) Slidex test for methicillin-resistant *Staphylococcus aureus* (MRSA) detection, using sensitized latex particles to react with MRSA, resulting in visible agglutination, and g) biochemical properties as assessed by the API20 ΝΕ (BioMerieux) microsystem.

Finally, identification of *Ps. aeruginosa, Klebsiella pneumoniae, Acinetobacter baumannii* and *S. aureus* based on microbes’ biochemical properties was conducted on the autoanalyzer Vitek 2, on marked cards for GN and Gram-positive (GP), after adding a bacterial suspension in saline in 0.5 MacFarland.

### 2.3. Drug Resistance Definitions

Multi-Drug Resistance (MDR) was defined as non-susceptibility to at least one agent in three or more antimicrobial categories. Extensive Drug Resistance (XDR) was defined as non-susceptibility to at least one agent in all but two or fewer antimicrobial categories (i.e., bacterial isolates remain susceptible to only one or two categories) [8].

### 2.4. Antibiotic Sensitivity Assessment

Antibiotic sensitivity was examined with two methods: (a) a Vitek 2 (Biomerieux) auto-analyzer for assessing Minimum Inhibitory Concentration (MIC) and (b) the E-test (Biomerieux) method.

All GN bacteria were examined in a Vitek 2 autoanalyzer for a number of antibiotics. Finally, and according to ECDC/CDC expert proposala [8], for *Klebsiella pneumoniae* specimens, an E-test was used for the antibiotics chloramphenicol, fosfomycin, tetracycline and colistin in order to characterize them as multidrug-resistant (MDR) or extensively drug-resistant (XDR). For GP pathogens, a Vitek 2 autoanalyzer was also used.

### 2.5. Statistical Analysis

Descriptive statistics were used. Due to the lack of normality in the distribution as assessed by the Kolmogorov–Smirnov test, for differences between groups the Kruskal–Wallis test was used. The statistical significance point was set at *p* < 0.05. SPSS v. 15.0 software was used for data analysis (IBM SPSS for Windows, Version 15.0.).

## 3. Results

### 3.1. Sampling Location

In total, 101 samples were collected. The distribution of air sampling locations is displayed in Table 1.

### 3.2. Environmental Parameters in the Four Settings

In the ICU, sampling was performed in the middle of the central hall and in the middle of two isolated rooms. Air renewal was performed 5–6 times/min through HEPA filters. No negative pressure ventilation was used; however, optimal hygiene practices were applied. The temperature ranged between 23–26 °C. Access to visitors was limited.

In the IMW and SW, sampling was conducted in examination rooms and patient wards. The temperature ranged between 23 °C (winter) and −26 °C (summer). Ventilation through open windows was achieved during spring and summer time. In these departments, access to visitors is extended beyond visiting hours.

In the neonatal unit the temperature was stable at 25 °C. Access to visitors was limited.

### 3.3. Identification of Microbes

Out of 101 total air samples from the four clinics, the following GN and GP microbes were isolated, as seen in Table 2.

As seen in Table 2, the highest proportion of the isolated pathogens, i.e., 26 GN strains and 59 GP, originated from the ICU, while the NU was the least colonized, with 3 GN and 15 GP microbes.

### 3.4. Microbial Load

The highest mean total microbial load was observed in the IMW (689/m^3^), followed by the SW (596 cfu/m^3^), the NU (509 cfu/m^3^) and finally the ICU (353 cfu/m^3^). This difference was statistically significant (Kruskal–Wallis analysis: chi-squred = 12.905, *p* = 0.005). 

Regarding the load of GN microorganisms, this was in general very low, with no statistically significant difference between departments (*p* = 0.471). The highest load was observed in the IMW (4.16 cfu/m^3^), followed by the ICU (1.14 cfu/m^3^), the SW (0.83 cfu/m^3^), and the NU (0.81 cfu/m^3^). Likewise, the load of *Enterococcus* spp. did not differ between the four departments. On the contrary, the microbial load of *Staphylococcus* spp. strains differed between departments, with the ICU having the highest mean value (245 cfu/m^3^), followed by the surgical ward (171 cfu/m^3^), the internal medicine ward (130 cfu/m^3^) and, finally, the neonatal unit (41.8 cfu/m^3^) (*p* = 0.018).

### 3.5. Comparison of Seasonal Variations in Airborne Pathogens, throughout the Year

**Figure 1:**
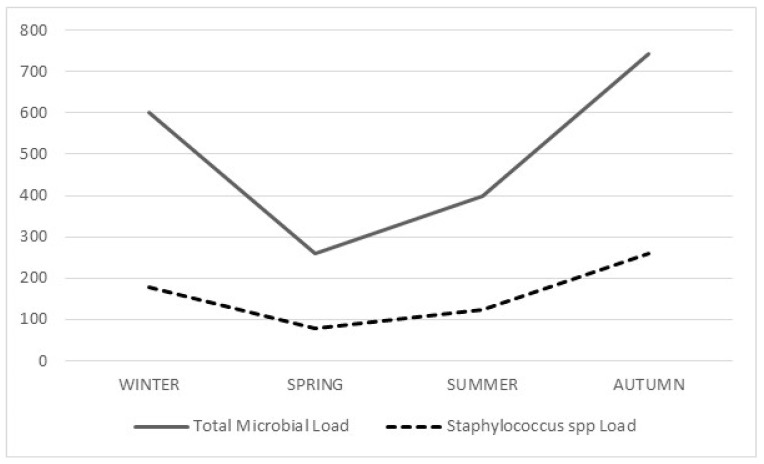
Seasonal variability of total microbial load and of *Staphylococcus* spp.

Microbial load varied between seasons. More specifically, total microbial load was higher in autumn (742 cfu/m^3^), followed by winter (599.9 cfu/m^3^), summer (399.8 cfu/m^3^) and finally spring (261.4 cfu/m^3^). The same pattern was observed in the seasonal variability of *Staphylococcus* load, which was higher in autumn in comparison to the other seasons (260.9 cfu/m^3^), followed by winter (177.4 cfu/m^3^), summer (123.7 cfu/m) and spring (79.5 cfu/m^3^) (Figure 1).

Likewise, *Enterococcus* spp. load was higher in autumn (20.1 cfu/m^3^), but it was followed by spring (3.4 cfu/m^3^), winter (1.9 cfu/m^3^) and summer (0.7 cfu/m^3^).

**Figure 2:**
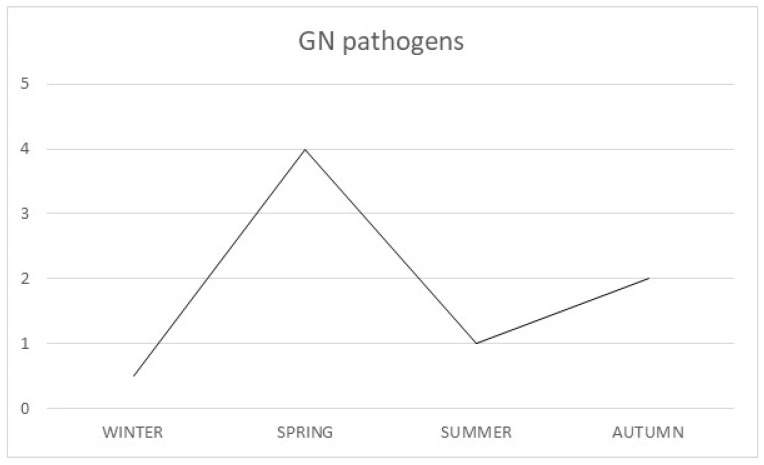
Seasonal variability of GN bacteria.

In contrast, the microbial load of GN pathogens was higher in spring (4.41 cfu/m^3^), followed by autumn (1.68 cfu/m^3^), summer (0.92 cfu/m^3^) and finally winter (0.44 cfu/m^3^). Regarding *Ps. aeruginosa,* no strains were identified in winter and autumn, while loads in spring and summer were almost identical (0.14 cfu/m^3^ and 0.13 cfu/m^3^ respectively) (Figure 2).

### 3.6. Drug Resistance of the Microbes, Isolated from the Inanimate Environment Samples

The distribution of MDR or XDR strains in the four departments is displayed in detail in Table 3, while Table 4, Table 5, Table 6 and Table 7 display the sensitivity test results that were obtained, resulting in the characterization of isolates either as MDR or XDR. As seen, most of the identified MDR/XDR strains derived from the ICU, 80% of which were GN. Regarding XDR strains, two GN, both *A. baumanii,* were detected: one in the ICU and one in the surgical ward.

## 4. Discussion

The present study examined the presence of microbial species and their load in the air of four different departments in a tertiary hospital, assessing at the same time their seasonal variability and their drug resistance. As previously reported in other hospital settings, *Staphylococcus* spp. was the most frequently isolated genus [9,10]. Indeed, and in accordance with previous studies, approximately 2/3 of microorganisms are GP (i.e., cocci and bacilli) while the rest consist of GN pathogens and fungi [4]. It is already known that GP microbes survive longer in aerosolized conditions and, in case of *S. aureus*, this is attributed to the fact that its cell wall is rich in peptidoglycane.

In our setting, indoor air was colonized with a significant number of GN and not exclusively with GP pathogens, as reported previously in literature [9,11]. Indeed, Aydin-Cakir et al. [11], during a 3-month sampling period in two hospitals in Izmir, Turkey, reported that the majority of the isolated bacteria in patient wards and corridors were *Staphylococcus* sp., followed by other GP pathogens. More specifically, 49/54 and 35/42 isolates were obtained from hospital 1 and hospital 2, respectively. Of these 84 Staphylococcus isolates, 42.86% were identified as S. aureus. Additionally, in another study from Portugal, the most frequent phenotype (88%) in all indoor environments was airborne Gram-positive cocci, with Staphylococcus (51%) and Micrococcus (37%) being the more dominant types [12]. In our samples, although the majority consisted of GP pathogens, a significant number of GN were isolated, originating mainly from the ICU.

Higher microbial load was observed in the IMW and SW, while in the NU and ICU the measured load was lower. In a study conducted in Ethiopia [13], which compared concentrations of bacteria and fungi on hospital air, it was demonstrated that higher bacterial concentrations were measured in the maternity ward, followed by the medical and surgical wards. Still, overall concentrations were very high, exceeding 2000 cfu/m3. As is known from the literature, in hospitals, as well as in other community places, the number of people visiting a place and the types of their activities as well as the frequency of cleaning are all associated with levels of bioaerosols [14]. Additionally, factors related to architectural design, like number and location of ventilation points, window positions, and room height are also important [14]. This was confirmed in our center, where ICU and NU are restricted areas with limited visiting access, contrarily to IMW and SW, where access to patients is easier.

The majority of isolated strains derived from the ICU. This microbial contamination of air can be an additional hazard for ICU-acquired infection, which adds to those previously reported in the literature, estimating the prevalence rate of infections in the ICU at 51% globally. In Europe, these rates range between 49% and 56.4% [15].

Seasonal variation is another finding in our study worth mentioning. As mentioned, in our center, measured GN load was higher in spring and GP load was higher in autumn, attributable probably to the climatologic conditions; however, this association was not explored. A previous study of indoor environment showed that peak level of indoor bacteria was measured in spring and the lowest level in summer; however, in this study, which was conducted in the indoor environment of houses in Denmark, the difference between GP and GN bacteria was not studied. The authors concluded that the season significantly affects indoor microbial exposure, and temperature, relative humidity, and air exchange rates are important factors that need to be taken into consideration [16].

The isolation of four different drug-resistant pathogens in our samples confirms the previous findings and raises concerns about patient safety, especially when taking into consideration that Greece has very high resistance rates regarding antibiotics [17,18]. Drug-resistant isolates were detected in all departments and involved *S. aureus*, *A. baumanii* and *K. pneumoniae*, *Enterococcus* spp. Mirhoseini et al., in their recent study, reported a high frequency of *A. baumanii* and *Staphylococcus* spp. [19]. A recent study of air samples in Iran, aiming to study the occurrence and characteristics of S. aureus isolates from different sources in ICUs (air, patients, healthcare workers and surfaces), reported 196 colonies in 42 air samples, out of which three isolates of *S. aureus* were detected, one of them resistant to erythromicin, one resistant to oxacillin and one to both antibiotics [20].

Regarding the IMW environment, not only was the highest microbial load measured, but two MRSA strains were also detected, while only one GN strain was detected. Likewise, in the SW, the vast majority were GP bacteria, which is in line with older studies of European origin, which also demonstrated that GP cocci are the most commonly found bacteria in indoor air environments [21].

NU had the least-colonized environment, as previously reported in the literature [22], which probably can be attributed not only to the limited access but also to the nature of the patients (i.e., newborns of low birth weight) with a limited load of cough droplets.

In conclusion, hospital air was contaminated with both GP and GN microorganisms. This contamination differed between settings and was characterized by seasonal variability. The isolation of MDR and XDR pathogens highlights the need for preventive measures and actions in order to inhibit the transmission of hospital-acquired infections among vulnerable patients.

## Figures and Tables

**Table 1:** Location of air sampling.

Location	Central Hall	Isolated Rooms	Examination Room	Ward	Total Number of Samples
ICU	12	24			36
Surgical Ward			12	12	24
Internal Medicine Ward			12	13	25
Neonatal Unit	16				16

**Table 2:** Number of isolates in each department.

	Number of Isolates				
	ICU	IMW	SW	NU	Total
***GN (total)***	**26**	**9**	**6**	**3**	**44**
*Klebsiella pneumoniae*	6	0	0	0	6
*Acinetobacter baumanii*	2	0	1	0	3
*Pseudomonas aeruginosa*	1	1	0	0	2
*Pseudomonas stutzeri*	1	0	1	0	2
*Pseudomonas oryzihabitans*	6	3	1	2	12
*Pseudomonas fluorescens*	1	1	1	1	4
*Enterobacter cloacae*	1	0	1	0	2
*Pantoea agglomerans*	1	3	1	0	5
*Escherichia coli*	7	1	0	0	8
***GP (total)***	**58**	**41**	**37**	**22**	**158**
*Staphylococcus* spp.	36	25	24	15	100
*Staphylococcus aureus*	2	5	1	1	9
*Enterococcus* spp.	22	16	13	7	58

**Table 3:** Distribution of MDR and XDR Isolates Among the four Departments.

	MDR	XDR	TOTAL
ICU	*K. pneumoniae* (*n* = 2) *A. baumanii* (*n* = 1) *Enterococcus* spp. (*n* = 1)	*A. baumanii* (*n* = 1)	5
IMW	*S. aureus* (*n* = 2)		2
SW	*S. aureus* (*n* = 1)	*A. baumanii* (*n* = 1)	2
NU	*Enterococcus* spp. (*n* = 1)		1

**Table 4:** Results of the Sensitivity Tests for MDR Kl. pneumoniae.

		1	2
Department		ICU	ICU
Location		Isolated room	Isolated room
Antibiotics	Aminoglycosides	R	R
	Antipseudomonal penicillins and b-lactamase inhibitors	R	R
	Carbapenems	R	R
	Non-extended spectrum cephalosporins, 1st and 2nd generation cephalosporins	R	R
	Extended-spectrum cephalosporins; 3rd and 4th generation cephalosporins	R	R
	Cephamycins	R	R
	Fluoroquinolones	R	R
	Folate pathway inhibitors	R	R
	Glycylcyclines	S	S
	Monobactams	R	R
	Penicillins	R	R
	Penicillins + b-lactamase inhibitors	R	R
	Phenicols	S	S
	Phosphonic acids	S	S
	Polymyxins	S	S
	Tetracyclines	S	S
Resistance type		MDR	MDR

**Table 5:** Results of Sensitivity Tests for MDR and XDR A. baumanii.

		1	2	3
Department		ICU	ICU	SW
Location		Isolated room	Central hall	Patient room
Antibiotics	Aminoglycosides,	R	R	R
	Antipseudomonal carbapenems	R	S	R
	Antipseudomonal fluoroquinolones	R	R	R
	Antipseudomonal penicillins and β-lactamase inhibitors	R	R	R
	Extended-spectrum cephalosporins	R	R	R
	Folate pathway inhibitors	S	S	R
	Penicillins + β-lactamase inhibitors	R	S	S
	Polymyxins	S	S	S
	Tetracyclines	R	R	R
Resistance type		XDR	MDR	XDR

**Table 6:** Results of Sensitivity Tests for MDR St. aureus.

	1	2	3
Department	IMW	IMW	SW
Location	Examination room	Patient room	Patient room
Aminoglycosides	S	S	S
Anti-MRSA cephalosporins	R	R	R
Anti-staphylococcal β-lactams (or cephamycins)	R	R	R
Fluoroquinolones	R	R	S
Folate pathway inhibitors	S	S	S
Glycopeptides	S	S	S
Glycylcyclines	S	S	S
Lincosamides	R	R	R
Lipopeptides	S	S	S
Macrolides	R	R	R
Oxazolidinones	S	S	S
Phenicols	S	S	S
Phosphonic acids	S	S	S
Streptogramins	S	S	S
Tetracyclines.	S	S	S
Type of resistance	MDR	MDR	MDR

**Table 7:** Results of Sensitivity Tests for MDR Enterococcus spp.

		1	2
Department		ICU	NU
Location		Isolated room	Central Hall
Antibiotics	Aminoglycosides (except streptomycin)	R	R
	Streptomycin	S	S
	Carbapenems	S	S
	Fluoroquinolones	R	R
	Glycopeptides	S	S
	Glycylcyclines	S	S
	Lipopeptides	S	S
	Oxazolidinones	S	S
	Penicillins	R	R
	Streptogramins	R	R
	Tetracycline	S	S
	Type of resistance	MDR	MDR
